# Assessing the methods needed for improved dengue mapping: a SWOT analysis

**DOI:** 10.11604/pamj.2014.17.289.3435

**Published:** 2014-04-16

**Authors:** David Frost Attaway, Kathryn H Jacobsen, Allan Falconer, Germana Manca, Nigel M Waters

**Affiliations:** 1 Department of Geography and GeoInformation Science, George Mason University, USA; 2 Department of Global and Community Health, George Mason University, USA; 3 Department of Geography and Director, GIS Center of Excellence, George Mason University, USA; 4 Institute of Public Health, Faculty of Medicine, University of Calgary, Canada

**Keywords:** dengue, geographic information systems, climate change, developing countries, medical geography

## Abstract

**Introduction:**

Dengue fever, a mosquito-borne viral infection, is a growing threat to human health in tropical and subtropical areas worldwide. There is a demand from public officials for maps that capture the current distribution of dengue and maps that analyze risk factors to predict the future burden of disease.

**Methods:**

To identify relevant articles, we searched Google Scholar, PubMed, BioMed Central, and WHOLIS (World Health Organization Library Database) for published articles with a specific set of dengue criteria between January 2002 and July 2013.

**Results:**

After evaluating the currently available dengue models, we identified four key barriers to the creation of high-quality dengue maps: (1) data limitations related to the expense of diagnosing and reporting dengue cases in places where health information systems are underdeveloped; (2) issues related to the use of socioeconomic proxies in places with limited dengue incidence data; (3) mosquito ranges which may be changing as a result of climate changes; and (4) the challenges of mapping dengue events at a variety of scales.

**Conclusion:**

An ideal dengue map will present endemic and epidemic dengue information from both rural and urban areas. Overcoming the current barriers requires expanded collaboration and data sharing by geographers, epidemiologists, and entomologists. Enhanced mapping techniques would allow for improved visualizations of dengue rates and risks.

## Introduction

Dengue fever (DF) is a mosquito-borne viral infection that causes high fevers and joint pain in the residents of at least 100 countries in Asia, Africa, the Pacific, the Americas, and the Caribbean. Mosquitoes (*Aedes aegypti and Aedes albopictus*) can become infected with any one of the four dengue virus strains when they take blood meals from humans infected with dengue; susceptible humans are then infected by these viral-infected mosquitoes. Some people with DF develop dengue hemorrhagic fever (DHF), which can be fatal.

Although many published studies have examined the behavioral and environmental risk factors for DF as well as the rates of human infection during outbreaks, a definitive methodology for mapping the incidence of this disease has yet to be established. These methodological deficiencies and gaps in the available data have limited the ability of geographers and other researchers to create effective visualizations for DF. An improved set of data and more effective mapping tools for the creation of local and regional dengue maps would facilitate communication about health risks among public health officials, healthcare providers, policymakers, and the public.

## Methods

To identify relevant articles, we searched Google Scholar, PubMed, BioMed Central, and WHOLIS (World Health Organization Library Database) for articles published between January 2002 and July 2013. The following search terns were used: (dengue OR dengue fever) AND (spatial analysis OR geospatial analysis OR GIS OR geographic information systems)]. No language restrictions were imposed. In total, 19 articles were selected for inclusion in the analysis.

To be included in the data extraction table, the articles had to (a) be published in or after 2002, (b) present a spatial analysis of dengue fever or *Aedes aegypti* mosquitoes, (c) incorporate qualitative or quantitative analytic techniques or a combination of both, and (d) use real-life rather than hypothetical data. The eligible articles represent data from a variety of world regions and model DF distribution using a variety of data sources and analytical techniques. This diversity of source material provided a solid evidentiary foundation from which to evaluate the current limitations for dengue visualization.

**Figure 1:**
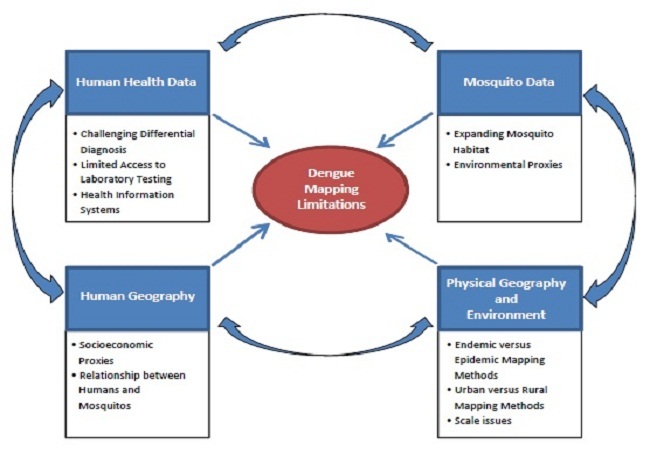
Summary of dengue mapping limitations

This paper categorizes the current barriers to the creation of high-quality maps of dengue risk at the local, national, and global levels (Figure 1). We begin by characterizing current data and mapping limitations as a first step toward overcoming the barriers to accurate visualizations of dengue rates and risks. We group these observations into four themes: (1) human health data limitations, (2) human geography issues, (3) mosquito data limitations, and (4) physical geography and environmental limitations. These four fundamental elements encompass critical issues for a successful Geographic Information System (GIS) for mapping dengue: appropriate mapping methods use valid and complete disease, vector, and environmental data. We further classify our observations by identifying sub-themes within each of the four main categories, which are presented in the Review section below. Finally, we use a SWOT analysis approach to clarify the strengths, weaknesses, opportunities, and threats to improved dengue mapping (Table 2) [1, 2].

## Results

Identifying barriers to dengue mapping is a necessary first step in improving the visualization of dengue. Each of the four key factors must be considered independently and in relationship with the other issues if the goal is to identify strengths and weaknesses of current models and opportunities to create better maps in the future.

**Human health data**. The ability to create useful maps of those areas of the world that are at risk of dengue transmission is dependent on the availability of accurate epidemiological data from those areas. Although dengue primarily affects low and middle income countries (LMICs), few LMICs have effective systems in place for dengue diagnosis and reporting. These human health data limitations exist for a variety of interrelated reasons.

**Challenging differential diagnosis**. Dengue fever symptoms tend to be clinically indistinguishable from many other tropical febrile infections. In many lower-income areas, patients with fevers are presumptively treated with anti-malarial medications, and those who fail to respond to anti-malarial medications or antibiotics are presumed to have a viral infection [3]. In malaria-endemic countries, over-diagnosis of malaria based on clinical symptoms rather than laboratory testing may be common [3], and presumptive diagnoses of malaria may cause the undercounting of cases of other febrile illnesses, including dengue. Since there are almost no effective treatments available for acute viral infections, there is often no clinical reason to conduct laboratory tests to determine the causative agent.

**Limited access to laboratory testing**. Dengue testing is somewhat more complicated than testing for many other viral infections [4]. The IgM (immunoglobulin M) levels in the blood that are markers of acute infection may not be detectable until several days after the onset of symptoms and then remain elevated for 2 to 3 months after the initial illness. Thus, a negative ELISA (enzyme-linked immunosorbent assay) IgM test in a patient with acute symptoms may not be proof that dengue is not the causative agent, and a positive ELISA in a person who has had a previous febrile illness in the past several months cannot be considered proof that dengue is the cause of the current fever. Another challenge to accurate diagnosis is the cross-reactivity of dengue with other flaviviruses, such as West Nile virus or yellow fever, which may cause false positive results. Because of these challenges, and because there are four different dengue virus strains, the results of serological (IgM) tests may need to be confirmed by molecular methods or isolation of the virus. Consequently, testing for dengue is an expensive proposition when compared to malaria, which can be diagnosed with just a microscope, or to viruses like HIV and yellow fever for which accurate rapid diagnostic tests are available. Even if health officials identified increased dengue surveillance as a priority in their countries, the costs of initial and confirmatory testing is in many instances prohibitive to patients and to health systems [5].

Health Information Systems. Maps are only as good as the data they are displaying, and an accurate global map of dengue risk is dependent on having at least some reasonably valid statistics from every country affected by endemic and/or epidemic dengue. At present, many countries have no official statistics for the incidence of dengue, even if they may have a presumed high burden of infection. Many countries where dengue is endemic or epidemic lack the resources to support robust health information systems (HIS) and extensive surveillance. A good HIS requires computer infrastructure, including internet connectivity; the ability to protect confidential electronic patient records; electronic medical records (EMRs) that are linked across hospitals, clinics, pharmacies, and other providers; communication between clinicians, laboratory specialists, public health officers; and a cadre of highly-trained data managers and analysts. Ideally, a strong HIS will include active surveillance in which public health authorities reach out to local clinicians to solicit case reports rather than merely relying on a passive surveillance system in which public health officials wait for healthcare providers to take the initiative to report a case. Passive surveillance systems may miss the majority of dengue cases, and even active surveillance systems face under-reporting of cases [5, 6].

The lack of infrastructure and the personnel and technology costs associated with establishing and operating an effective HIS are significant barriers for LMICs which must generally prioritize urgent patient care needs over the implementation of longer-term public health activities. Even if subsidies are available to help offset the costs of software licenses and equipment, the costs of training and supporting epidemiologists and GIS analysts would be substantial, especially if the goal is to run sophisticated geospatial analyses [7–10].

The advent of internet-based data collection systems may provide an opportunity for dengue researchers and others with related interests to share data that may allow for better mapping of dengue risk. For example, MosquitoMap is an online database within VectorMap that compiles geospatially-referenced mosquito data [11]. The repository includes information from the Walter Reed Biosystematics Unit, the School of Integrative Biology at the University of Queensland, the U.S. Army Medical Research Unit-Kenya (USAMRU-K) and Kenya Medical Research Institute (KEMRI), and other reporting agencies [11], and includes, among other resources, maps of the environments that are suitable for Aedes aegypti [12]. However, while geospatial mosquito data may contribute to predictive models of dengue risk, mosquito range data alone are not sufficient for mapping actual dengue incidence and prevalence. The data from MosquitoMap must be seen as supplemental to accurate data on human dengue fever cases and not as a substitute for human data.

The collection and dissemination of additional spatially-linked data on human dengue cases will require careful attention to maintaining the confidentiality of personal health and other data and protecting the privacy of individuals. Aggregating data to a local scale can protect sensitive medical records while allowing for greater map sensitivity.

**Human geography**. Human geography, which describes the socioeconomic and cultural factors that influence how various populations interact internally, with other populations, and with the environment, is an important tool for medical geographers and other researchers. Dengue researchers must understand how changes in economic development, international and rural/urban travel, and human patterns of interaction relate to dengue epidemiology. Several human geography issues contribute to dengue mapping limitations.

**Socioeconomic proxies**. When dengue incidence rates are not available, proxy variables may be used to identify populations likely to be at risk. Proxies infer the likelihood of incident dengue occurring based on socioeconomic, demographic, and/or environmental trends rather than data from confirmed or suspected cases. As a result, the estimates may significantly underestimate or overestimate the actual dengue risk in various populations. For example, economic proxies, such as the relative poverty of neighborhoods within a particular urban area, may be used since lower-income areas tend to have greater dengue risk [13, 14].

Records of human behavior such as travel records [3, 5, 15] are also sometimes used as proxies for exposure to dengue. Studies tracking human movement that might bring susceptible individuals into contact with *Aedes*-inhabited zones have used GPS data-loggers, but the data obtained from a small sample of individuals in one town are not necessarily generalizable to an entire region [16]. Thus, the current social and environmental proxies for dengue have some “scale” limitations: measures like GDP and GNI are relevant only at the national level while data collected at the neighborhood level, such as one city or one part of a city within a country, are not able to generalize to a larger area.

**Relationship between humans and mosquitoes**. A variety of factors may change the vector density of *Aedes* mosquitoes in a particular area, including climate change (global warming) and human activities such as poor water and waste management, which can create standing pools of water that can serve as breeding sites. Human behaviors also influence the rate of infection of mosquitoes with dengue virus: increasing human population density (especially when associated with poor environmental management from unplanned urbanization), the growth of international trade and tourism (which facilitates the spread of dengue to new places), and changes in public health policies and practices (including those related to environmental management and vector control) [17–20]. Additionally, hyperendemicity with more than one strain of dengue in some places puts people at risk from multiple strains [21], a situation that is best understood when it can be visualized. Because both human and mosquito population density and behavior contribute to dengue incidence and spread, data from both populations must be incorporated into dengue mapping models.

**Mosquito data**. The expected expansion of mosquito habitats due to climate change means that current maps that show the ranges of mosquito species may soon be obsolete. Changes in mosquito ranges may limit the ability of existing mosquito habitat data to serve as a proxy for dengue endemicity data, because the mosquito data will no longer be up-to-date and relevant.

**Expanding mosquito habitat**. Given the relationship between mosquito habitat and climate, the range of many mosquitoes, including the *Aedes* species, is expected to expand with climate change, exposing more people to dengue [22, 23]. Prediction models suggest that global warming may increase the latitudinal range, the altitudinal range, and the duration of the transmission season for *Aedes* mosquitoes [24, 25]. Several dengue maps have attempted to translate Aedes range models to predictions of changes in dengue distribution, but all admit serious limitations due to the complexity of the analysis and the limited accuracy and completeness of data for model parameters [26–28]. If the range of mosquitoes changes significantly in coming years, new dengue maps will have to be created often in order to be of use to health policymakers and practitioners.

**Environmental proxies**. If the range of *Aedes*. mosquitoes is demonstrated to be changing more frequently than in the past, mosquito maps may no longer be sufficient proxies for dengue infection. Many of the published projections of dengue endemicity from recent years were created from map layers showing temperature, rainfall, and land features that are meant to identify areas that are biologically suitable *Aedes* habitats [22, 24, 29]. If these parameters can no longer be measured or estimated with accuracy due to climate change, estimates of dengue range will have to depend on other forms of proxy data.

**Physical geography and the environment**. Dengue is often considered to be primarily an urban infection, in large part because *Aedes* mosquitoes thrive in urban environments, but dengue also occurs in rural areas [30–32]. The need for endemic and epidemic maps that provide reasonable estimates for both rural and urban areas requires addressing several physical (environmental) geography concerns. Additionally, most current models display only the presence or absence of dengue, and there is a need for a more nuanced portrayal of levels of risk and levels of the burden of disease from dengue within affected areas.

**Endemic versus epidemic mapping methods**. Dengue maps tend to fall into two categories. Endemic maps show which places experience dengue transmission during at least some months in a typical year, usually presenting estimates of dengue presence at the country or regional level [3, 5]. Epidemic maps display outbreak information from places where the infection rates are considerably higher than normal, and tend to show data specific to neighborhoods, cities, or districts [33–35].

Public health officials need geographic information about both endemic and epidemic dengue. The maps included in the U.S. Centers for Disease Control and Prevention's (CDC) Yellow Book- the travel vaccine guidelines are re-issued every two years- show which countries are considered endemic for dengue [35]. This book is widely used by clinicians who provide travel advice. In contrast, the DengueMap project updates an online map every hour to show the precise locations of cases of dengue that have been reported by public health officials, newspapers, and internet sources such as ProMED mail, which is sponsored by the International Society for Infectious Diseases and allows anyone in the world to submit brief case reports about emerging epidemics. These approaches provide complementary information for use in decision-making about public health and personal health, but it is difficult to provide one map that captures both ways of viewing risk.

**Urban versus rural mapping methods**. Dengue mapping has focused mainly on studies of urban areas, in part because most dengue infection surveillance is conducted in high-density population areas. Urban dengue maps factor in considerations of human and mosquito population density, demographic and environmental factors, and vector control initiatives. Rural areas have lower population densities, different demographic and environmental considerations and data availability, and limited vector control efforts. In particular, urban and rural areas have distinct energy balances, temperatures, humidity levels, and storm runoff (and standing water) patterns [36]. As a result of these special environmental considerations, current mapping techniques require different approaches in urban and rural areas. The lack of human and mosquito data for rural areas often requires greater dependence on temperature, precipitation, landforms, and other purely environmental data and estimates, and demands a small scale approach even though more detailed scales would be beneficial to map users [37]. Dengue maps would be more useful to policymakers and others if they provided a more complete analysis of dengue distribution and risk factors across the spectrum of population density.

**Scale Issues**. Depending on the availability of geographically referenced data, effective mapping should include both temporal and scale considerations. Dengue maps highlight the scale differences between global, regional, and local mapping. Mapping of dengue endemicity on the global scale is usually coarse because global climate datasets have relatively coarse spatial resolution [29]. Recent models suggest that mapping dengue at the country level underrepresents dengue occurrence [3, 5, 38, 39]. At the same time, local models with site-specific data are often limited in generalizability to locations with similar climate, geography, urbanization, and various human geography factors, and may be so specific to one place that they cannot easily be applied to other regions [23, 24]. Comprehensive dengue mapping projects will need to develop methods for integrating global climate scenario-based analysis together with local demographic and environmental factors, including those from both rural and urban areas.

## Discussion

A SWOT analysis, though traditionally recognized as a tool to evaluate businesses and projects, provides valuable insight into methods necessary for improved dengue mapping. SWOT uses evaluations of current performance to develop strategic plans for improving future operations [25]. The four components of a SWOT analysis examine (1) the current strengths that should be maintained and built on, (2) the weaknesses that need to be addressed, (3) the opportunities that are available for moving toward more optimal function, and (4) the threats that may prevent progress from being made [2, 25]. SWOT provides a framework for evaluating the benefits and limitations of current models that should be considered as new techniques for advancing mapping and visualization are developed. Table 2 summarizes the SWOT factors for dengue mapping that were presented in the Results section. The SWOT analysis points toward actions that would allow for improved spatial analysis of dengue fever distributions and risks.

One major theme that emerges from the SWOT analysis is the critical need for better data collection and sharing. Improved data collection would require a commitment to global cooperation and allocation of appropriate resources to this project (which for cost savings could include many infectious diseases of global interest rather than focusing exclusively on dengue). Once more data are being collected using comparable methodologies, the creation of well-maintained and accessible cross-national data repositories will be incredibly helpful for advancing public health knowledge and for allowing geographers to develop, test, and compare methods for mapping dengue endemicity.

As better data sources are developed along with more robust software for integrating various types of data, there will be an opportunity for new models to incorporate a more diverse set of data about spatially-specific incidence, human demography and behavior, mosquito ranges, and other environmental factors, such as elevation and precipitation. An ideal multi-level model will be viable for local and regional analysis as well as for examinations of the global burden of dengue. Additionally, the model will be one that can be updated as often as new data become available, including live-time data uploaded during outbreaks.

## Conclusion

Understanding current data and mapping limitations is an important first step toward overcoming the barriers to accurate visualizations of dengue rates and risks. The development of new maps that inform public health policy and practice will require geographers, epidemiologists, and entomologists to commit to sharing resources and knowledge and to working together to build robust systems for data collection and dissemination, data analysis, and distribution of up-to-date maps. New technologies may then allow for the integration of multiple input variables across global, regional, and local scales and the creation of better dengue maps for use in public health decision-making.

## Additional material

**Annex 1 (PDF - 231 Kb)**: Summary of the 19 eligible dengue mapping articles

**Annex 2 (PDF - 177 Kb)**: SWOT analysis for improved dengue mapping
